# Research group as helpers due to the war in Ukraine: Focus group experiences of women researchers

**DOI:** 10.3389/fpsyt.2023.1139252

**Published:** 2023-03-01

**Authors:** Xenia Roszik-Volovik, Anna Paula Brandão, Nóra Kollárovics, Bernadett Frida Farkas, Eszter Frank-Bozóki, Lili Olga Horváth, Zsuzsa Kaló, Lan Anh Nguyen Luu, Judit Balazs

**Affiliations:** ^1^Department of Developmental and Clinical Child Psychology, Eötvös Loránd University, Budapest, Hungary; ^2^Doctoral School of Psychology, Eötvös Loránd University, Budapest, Hungary; ^3^Doctoral School of Mental Health Sciences, Semmelweis University, Budapest, Hungary; ^4^Nemzetközi Cseperedő Alapítvány (International Cseperedő Foundation), Budapest, Hungary; ^5^Department of Counselling and School Psychology, Institute of Psychology, Eötvös Loránd University, Budapest, Hungary; ^6^Institute of Intercultural Psychology and Education, Eötvös Loránd University, Budapest, Hungary; ^7^Department of Psychology, Oslo New University College, Oslo, Norway

**Keywords:** working with refugees, war in Ukraine, effects on helpers, volunteering, development of voluntary help, female research group

## Abstract

**Introduction:**

World Health Organization studies have shown that one in every five people who have experienced war or other conflicts suffers from a mental health disorder, the most vulnerable groups being children and women. According to international guidelines, mental health care should be made available immediately in the event of disaster. With the first influx of Ukrainian refugees to Hungary at the outbreak of the war, the Research Group of Childhood Mental Health at Eötvös Loránd University and Semmelweis University in Budapest immediately decided to help by transforming itself into a support group for refugee families. The members of the support group are all women. The aim of the present study is to explore the motivation behind the transformation of the research group and the help it provided. A further aim was to compare the group’s experiences with descriptions in the literature of impacts on helpers who work with refugees.

**Methods:**

The current paper reflects on the transformation from researchers to helpers and the effects of that transformation at group and individual level using the focus group method and consensual text analysis. The transformation of the support group necessitated the involvement of students, whose experiences are also examined.

**Results:**

We identified five main categories: context; the help recipients’ perspective; the personal level; the professional level; and the level between the personal and professional.

**Discussion:**

The analysis revealed the way in which the voluntary helping developed, the resulting difficulties, and coping options. Volunteering among Ukrainian refugees has both positive and negative psychological consequences. While stress and trauma threaten the psychological well-being of the helpers, positive aspects, such as flexibility and professional development, are also reported. Due to the strong motivation among group members and their experience in practical work, the all female research group was quickly able to transform itself into a support group.

## 1. Introduction

According to official statistics, more than six million refugees, most of them women and children, have fled Ukraine since the outbreak of the war (1, 2). The number of Ukrainian refugees in Hungary is estimated at more than half a million (2). As a general health response, mental health support programs and organizations have a crucial role to play. Those displaced by war are particularly vulnerable to mental health problems, with women and children at the greatest risk (3).

Refugees are typically exposed to physical threats and fear and must cope with the psychological consequences (3–5). Separation from home and the loss of loved ones are common experiences (3, 4, 6, 7), as are coping emotionally with separation from family members who have been left behind and constant anxiety for their safety (8–10). Women may have been separated from their husbands/partners and elderly parents, while children may be dealing with separation from, or mourning the loss of, fathers, grandparents, and in some cases mothers (10, 11). On arrival in the host country, refugees face the difficulties of adapting to their new circumstances and the challenge of language and cultural differences (4). Several studies have shown that young refugees are at increased risk of psychological distress and that there is a significantly higher prevalence of mental disorders among them compared to their peers in the host country (12–15). Systematic literature reviews have highlighted the prevalence of severe mental disorders among young people exposed to the stress of war, with between 19 and 54% suffering from posttraumatic stress disorder (PTSD) and between 3 and 30% experiencing depression (11, 16–20). One study of a population affected by the war in Ukraine showed that the loss of a loved one was one of the strongest predictors of developing PTSD (21). While financial security may reduce the risk of PTSD, this is not the case among refugees with children, as demonstrated in the Ukrainian population (21). As pointed out by the World Health Organization (WHO), reducing the risk factors may play a role in preventing the onset and recurrence of mental health disorders (3, 22).

In the context of PTSD, one protective factor is strengthening the relationship between individuals and the community (23, 24). Refugee workers have a huge role to play in this process. Helpers can be divided into two broad categories: paid helpers and volunteers. Both groups are exposed to psychological strain, burnout, and psychological and mental trauma (25). Pressure, forcedness, and emergency situations are key antecedents of refugee migration, while refugee assistance is organized in different stages (26). According to one study, the process of helping refugees involves: (1) noticing and recognizing distressing conditions and emergencies in which help is required; (2) taking responsibility; (3) knowing how to help; and (4) transforming knowledge into action (26). Empathic concern plays an important role in the first step (27). Although little research has been carried out into the process of taking responsibility, it seems to persist in the long term if the assisted refugees accept and pursue integration or assimilation into the majority society (26, 28). Effective assistance to refugees requires specific knowledge, for example knowledge of the traumas the refugees have faced, of working with trauma, and of cultural and linguistic specificities (26, 29).

While those who help refugees provide an invaluable service, it is also important to be aware of the potential vulnerability of the helpers themselves. Helpers typically experience psychological traumatization more often than PTSD (25). Helpers may be exposed to stressful situations that traumatize them, that they do not expect, and for which they cannot prepare in advance (30). McCann and Pearlman described how helpers may be exposed to traumatization in their empathic work with trauma survivors (31). Helpers may experience emotional overinvolvement, self-blame, shame, and guilt; prolonged anger and sadness at the patient’s victimization, loss of hope, pessimism, and overidentification with the patient, resulting in the recurrent recollection of the traumatizing content; unrealizable rescue fantasies; and patient rejection and avoidance in order to avoid the traumatizing content (32).

Volunteers are motivated by a number of factors. Studies have shown that volunteering can enhance feelings of self-satisfaction through altruism (33, 34). Different narratives and media content can also influence the emotions, which can affect motivation to volunteer, as pointed out by Karakayali (35) and Kleres (36). Two types of help are discussed in studies on the topic: (1) benevolent support, motivated by a desire to reduce the sufferings of others; and (2) social activism, which involves the underlying motivation of changing or supporting a particular social, legal, or political structure (37, 38). In the case of benevolent support, helpers are less concerned with underlying causes and injustice (37). Those who volunteer tend to be prompted by a combination of individual motives and personal interests that lead to the provision of help in many different ways (39). In the present study, our aim was to investigate these manifold motivations, the difficulties experienced, and the potential for improvement of the volunteering experience.

### 1.1. The Research Group of Childhood Mental Health at Eötvös Loránd University and Semmelweis University, Budapest

The Research Group of Childhood Mental Health at Eötvös Loránd University (ELTE) and Semmelweis University (SE), Budapest (hereinafter “the research group”), currently has 14 active members. The group conducts its research in the framework of the doctoral schools of ELTE and SE. The research group is led by Dr. Judit Balázs, a specialist in child, adolescent, and adult psychiatry and a professor at ELTE. The members of the research group who participated in the work are a psychologist and doctoral student at ELTE; a counselling psychologist and doctoral student at ELTE; a medical doctor and doctoral student at SE; a child psychiatrist and doctoral student at SE; and a clinical psychologist and doctoral student at ELTE.

The members of the group are involved in the following research areas, from the perspective of prevention and treatment: youth suicide and self-harm; neurodevelopmental disorders; quality of life; sub-threshold mental disorders; and mental health, illness, and quality of life among immigrants and refugees.

### 1.2. Activities related to the outbreak of the war

A detailed description of the research group’s activities is currently being prepared for publication. The brief summary below is necessary for an understanding of the research described in the present paper.

At the beginning of the war, the leader of the research group, Dr. Judit Balázs, suggested that the group might provide assistance to the refugees. Everyone agreed with the suggestion, and it was decided to organize playgroups for the children of Ukrainian refugee families to help them feel welcome and to alleviate their potential fears. Rather than providing professional (psychological/psychiatric) help, the first step was to create a small playful “island of peace,” mainly for children and their families.

The organization of the playgroups started in the first weeks of March 2022, immediately after the outbreak of the war, and members of the research group volunteered their time. Games and drawing activities were planned, along with other activities that did not require language skills. We were aware that the children who joined the playgroup would potentially be in a traumatized state and thus tried to listen and simply be present. In addition, we planned to have informal discussions with the parents during these playgroups, enabling the refugee families to meet each other, exchange experiences, and form social relationships. As psychologists and psychiatrists, we were also prepared to provide help, guidance, and advice for those with specific questions.

The adult refugees were predominantly mothers and grandmothers, with very few men. We communicated mostly in English, Russian, Ukrainian, and Hungarian. We also tended to rely on non-verbal communication with the children, playing together with them and smiling a lot.

On March 8, the first group was launched at ELTE, and two sessions were subsequently held each week. We created a Facebook group for the parents who had participated in the playgroups, aimed at: (1) making it easier for families to find one another; (2) advertising upcoming sessions; and (3) linking other events groups for children and families. The playgroups had closed by the end of May due to dwindling attendance, mainly because the children had started attending school and because an increasing number of refugee programs had begun to appear.

In parallel with the formation of the ELTE playgroup, we learned that many families, mostly Transcarpathian Hungarians, were being housed about 30 km from Budapest. Communication with these families was easier because they spoke Hungarian. They also had a different socioeconomic status from the families we had encountered in the ELTE playgroup. We visited the families twice a week, and once again the primary objective was to provide playful, developmental activities, football and recreational games, entertainment, support, and an atmosphere of acceptance. This program is still being run by members of the research group, who visit the families once a week.

A third project involved the Budapest Circus, which had found out (*via* a Facebook ad) that our group was organizing activities for refugee children. The circus opened its doors to more than a hundred children, most of whom were without their parents. By way of experiment, we included a psychological session in this project: we developed a four-week group session focusing on stress reduction and coping strategies, using elements of art therapy. We also involved an external supervisor and interpreters.

From mid-March, the three projects (the ELTE playgroup, visits to families accommodated outside the capital, and the Budapest Circus) were running in parallel. Due to the workload, our team divided into three groups, each responsible for one of the projects, although we continued to have joint meetings to exchange ideas and experiences. A core team of six members was formed, along with a core team Messenger group and Google group to facilitate correspondence and event organization.

The second project continues to operate on a voluntary basis among the families accommodated outside Budapest, although the goals have now changed. We are trying to implement social and psychological support alongside the games, while maintaining the nature of the group work with the children.

A total of 23 university students participated in the sessions, most of them MA students from the Department of Developmental and Clinical Child Psychology at ELTE’s Institute of Psychology. On one occasion, a Ukrainian-speaking international student joined the Budapest Circus program, and a Ukrainian-speaking international PhD student joined in several sessions of our ELTE playgroup project. We offered MA students the possibility to exchange their summer internship for participation in our projects. The students were invited to sign up on a Google Drive spreadsheet to indicate which project and how many sessions they would like to participate in. Their task was to be present, to engage with us, and to play with the children.

## 2. Aims

In the present paper, we reflect on the experiences of the research group and the students who helped the refugees. Our study focuses on lived experiences, with an emphasis on the following themes:

- What motivates people to help in a crisis? What motivations emerged at professional or personal level when the projects were launched?
- What experiences emerged in terms of the potential professional and personal development that can take place when helping people in crisis?
- What psychological risks and difficulties were experienced in the process of helping the refugees from Ukraine? What were the internal and external resources that helped and empowered the volunteers to overcome these difficulties?

## 3. Methods

### 3.1. Ethical permission

The study was carried out with the permission of the Research Ethics Committee of ELTE PPK (approval number: 2022/386). Participants gave their written informed consent after receiving verbal and written information. Participation in the study was voluntary and could be withdrawn at any time.

### 3.2. Participants

Participants in the study fell into two groups: (1) the core team, comprising members of the research group; and (2) MA psychology students from ELTE. The core team was made up of six people, all of them women. In terms of qualifications, the members of the core team were a child and youth psychiatrist; a child, youth, and adult psychiatrist; a general practitioner; a psychologist and doctoral student; a clinical psychologist; and a counselling psychologist. Six MA students participated in the focus group, all of them women.

### 3.3. Interview protocol: Focus group method

We chose to use the focus group method for our study, in which members of the group are given the opportunity to recall events together and reflect on one another’s views. Since the voluntary work was largely dependent on group formation and group functioning, it was important for us to have a space where group members could voice their individual experiences alongside the group experiences. The focus group method was the most appropriate for this (40).

The semi-structured set of questions focused on the experiences of the focus group participants. The interview themes were: (1) motivations for helping; (2) the emotional experiences of helping; (3) how helping refugees differed from giving help in other circumstances; and (4) resources that helped the volunteers to cope with the difficulties. The core team focus group was interviewed by a PhD student and an MA student from the Qualitative Psychology Research Group at ELTE. The core team focus group session lasted for 120 min, while the student focus group session lasted for 62 min. The sessions were video recorded with the consent of the participants, after which exact transcripts were prepared. The character counts of the transcripts were 79,649 (with spaces) for the core team focus group session; and 45,733 (with spaces) for the student session.

### 3.4. Content analysis group

Text analysis was carried out by five people. Three members of the text analysis team were core team members and two were external experts, and all of them volunteered as individuals, independently of the current core team. Two are bilingual and have an immigrant background.

### 3.5. Data analysis: Qualitative consensus method

The analysis was conducted from a postpositivist/constructivist epistemological inductive approach, using the consensual qualitative analysis method. According to this method, if qualitative data analysis is conducted under properly controlled conditions, the results can capture and reflect the reality of the data (41). Data analysis involves the intensive analysis of a small sample, with particular emphasis on putting experiences into context. Consensual analysis involves the following steps: (1) *the application of domains—*it is important to consider not only the focus group questions but also the themes introduced by the participants, as this will best reflect what the participants have to say. Each text analyser contributes individually, after which a consensus list is drawn up in a joint discussion; (2) *core ideas—*in the unified list, the quotes under each domain are summarized in a few key sentences. Individual group members again work individually, after which consensus is reached on the meanings of the domains in the context of a group discussion; and (3) *cross-analysis*—the core ideas are sorted into categories. Once again, everyone works alone initially, before consensus is reached through joint discussion. At each step, the consensus decisions are monitored by an independent auditor who adds their insights and corrects the process. In this way, categories are constructed *via* systematic and continuous deliberation, reflecting the complex experiences lived in context.

## 4. Results

We divided the experiences into five dynamically linked categories that build on one another. Each level stimulates the others, ultimately reflecting the complex facilitation processes and, within them, the nature and content of the facilitators’ burdens and how these difficulties are overcome.

**Figure 1:**
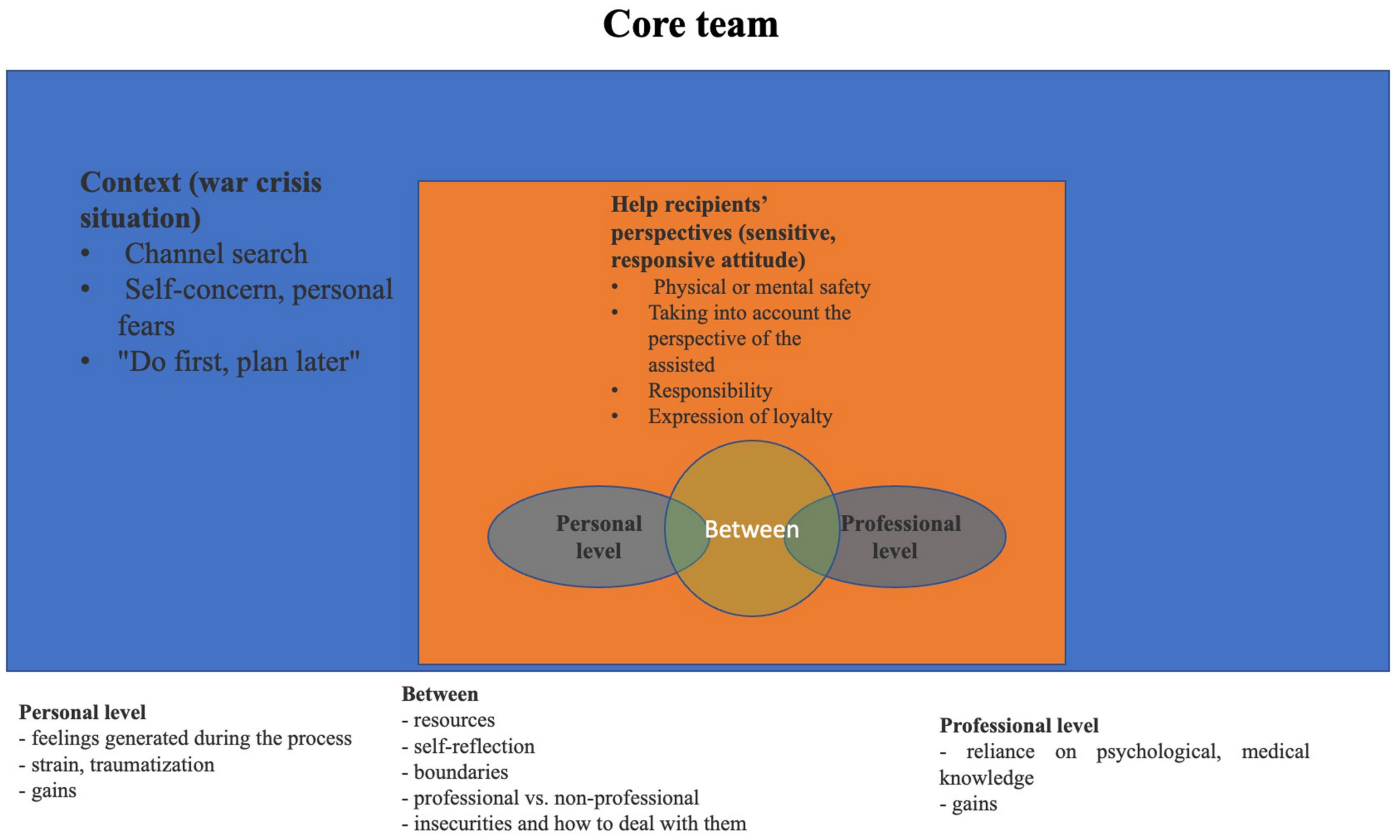
Results of the consensual content analysis—the categories established from the core team topics and the relationships among them.

**Figure 2:**
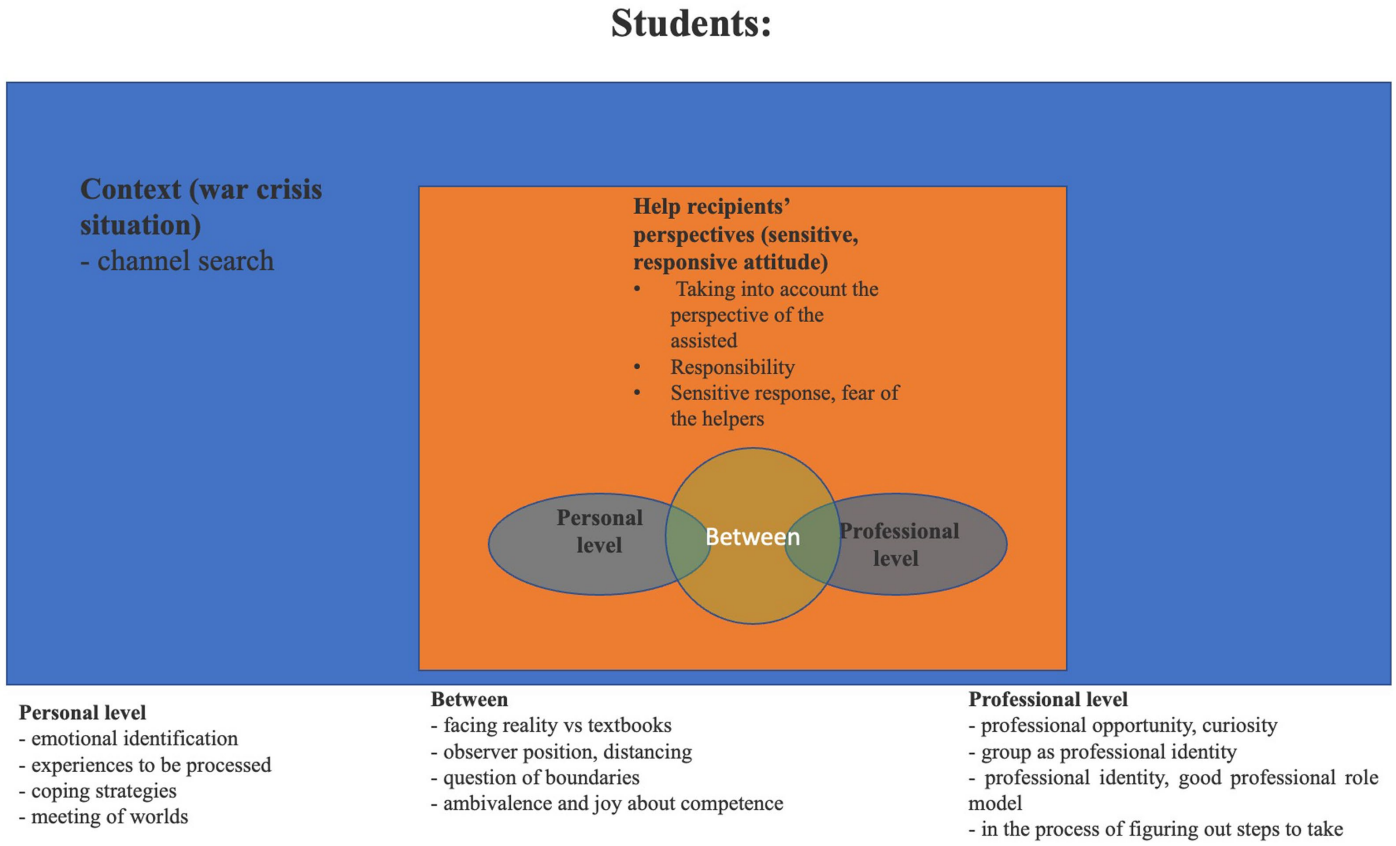
Results of the consensual content analysis—the categories established from the university students’ topics and the relationships among them.

The five categories are: (1) context; (2) the help recipients’ perspective (sensitive responsive attitude); (3) the personal level; (4) the professional level; and (5) the level between the personal and professional (Figures 1, 2).

### 4.1. Context

In this category, we selected the experiences of the core team members and students that were specifically related to the outbreak of war and that expressed the dynamics and experiences of the context of a war crisis. From the very first days of the war, an internal, growing sense of tension and helplessness was apparent among the would-be volunteer helpers in both groups. Both the core team members and the students were looking for a channel for the release of these growing tensions, which represented a significant pressure to start the process of helping.

…there was a very, very high tension inside me because I felt I had to do something, but it was very difficult to find channels for it without it being so chaotic… (core team member)

The students had also experienced the tension of “finding a channel,” and our research group’s initiative within the department proved to be a good opportunity for them:

I felt a bit helpless in that situation and I was very, very happy that there was such an initiative in the department. So, on the one hand, I had this desire to help, and I just couldn’t have found the means to do it myself. (student)

The outbreak of war gave rise to sensitivities, anxieties, and fears at a personal level, which motivated people to help. There was also a sense of identification with the refugees on the part of students and core team members, in the form of personal anxieties and fears that this problem could become theirs and that their way of life could change.

I think I share with the group, there was… we were afraid that […] if something happens, we should leave. I think when everything started […] I was very afraid of everything, like feeling that, OK, maybe I should leave […], and we talked about it, that we are all in the situation that we are all in danger, or not, and what is going to happen… (core team member)

…such a split thing […], because on the one hand I had such a very big anxiety for my children […]. And then, on top of that, the need to do something, and then such a wobble from one side to the other. (core team member)

In the increasing tension and crisis of the war, there was little time to plan. Among the helpers, action preceded detailed planning; it was important to provide assistance as soon as possible and to reduce internal tensions at several levels:

…it can’t be that I’m sitting here in Budapest, they’re coming there hungry, so it’s absolutely necessary to go there to help […] together we thought about it and looked for a direction in which we could help effectively. (core team member)

So that’s a little bit ahead of what we need to do and we’re still thinking about it, we’re coming together, now we’re trying to consider what we can do. (core team member)

The facilitation process was accompanied by a sense of “catching up with oneself”:

It’s permanently in there, as XY said, to educate ourselves, to help in a clever way, but somehow we are carried away by events. (core team member)

### 4.2. The help recipients’ perspective (sensitive, responsive attitude)

In the context of the emerging situation, the attitude of the facilitators was strongly influenced by the inclusion of the refugees’ perspective. The attitude of the helpers was essentially sensitive and responsive. By adopting the viewpoint of the recipients of the help, the helpers tried to provide continuous, empathic listening. They tried to protect the refugees from potential disappointment and to offer a caring presence. They avoided any kind of “exploitation” as a research group, at the same time fearing that others might take advantage of the refugees’ plight. This meant putting aside their everyday working attitudes as researchers:

…from a research perspective they are a treasure, but right now they shouldn’t be overloaded… I was very afraid for them, that no extra burden… Don’t overload them… (core team member)

…there was no preparation, I had to realize that there was a very pregnant woman there […] and actually it was a responsibility… a responsibility for them so that she could give birth safely. And I was very scared that she might be subject to discrimination or have a bad experience when she went into hospital, and she should be prepared, and so on and so forth, and so we prepared together, we bought her stuff together, so I just had to be constantly aware of practical things, which is quite far from our […] research work, and from my work as well… (core team member)

And I had this kind of protectiveness, so in addition to anger I had this kind of defensive feeling… (student)

In this situation, showing loyalty towards the refugees was inevitably expressed as one aspect of a sensitive attitude:

…with such a human presence, with compassion, expressing quite clearly which side I’m on, that I’m supportive […]. (core team member)

### 4.3. The personal level

During their encounters with the refugees, both the core team members and the students experienced many different emotions, which they had to deal with. They were encountering unfamiliar, incongruous, extraordinary situations. Helping the refugees involved a strong emotional involvement and engagement on the part of the helpers:

First two little boys and then other children got involved, starting to act out the war with Lego figures, and what the outcome would be. And I remember even now the gut cramp, that I don’t know what the outcome is going to be; they don’t know obviously, and so I’m cheering for something positive to happen. (student)

In one refugee shelter, a baby was born shortly after the family’s escape, which resonated emotionally with many people—both students and core team members:

For me, what really touched me was when I found out that a baby had been born… So, I don’t know, I was so shocked, like, oh my God, I don’t know… (student)

The forming of immediate, intense attachments with the refugees, followed by rapid separation and a lack of closure in the relationships, emerged as a difficulty at the emotional level among core team members:

It’s hardest in the summer, or the way the families are dwindling, and not being able to close that relationship or round it off, or not being able to say goodbye. (core team member)

At a personal level, situations in which the helper experienced tension or anger because of something the refugees had done proved to be extremely difficult emotionally for the helper:

Experiencing anger, …at the parents, the way they handled it, while it seemed that the activities were good for the children, and then being in shock that these are refugees, and then I’m angry with them […]. But at the same time there is obviously room for anger, but it’s a terrible feeling to experience that—How can you be angry at a refugee? (core team member)

The participants felt a profound sense of guilt at the idea of doing anything else when the priority was to help the refugees. Also, there were expectations about what needed to be done in the situation, once they had heard about the difficulties from the refugees:

…it’s not fair to do anything else […]. I had to leave after a month or so for an international EU meeting. […] And so I said to the other people there, how is it possible that we are talking about this? (core team member)

…I felt there that I couldn’t speak, not because I didn’t speak the language, but because I simply didn’t have the means to push this forward, when that was my job, and that was terrible for me. So, I’ve very rarely been in this situation, fortunately, for example at work, where I don’t know what to say when something is said, but it was also somehow an expectation in myself that this can’t happen, that I can’t. And then my strategy ended up being to say that it must be very difficult, and it’s very difficult to speak. (core team member)

There were also situations that were emotionally charged to such an extent that they had a longer-term impact and took longer to process, consistent with the phenomenon of psychological traumatization:

There was one time, by the way, when we came out like that, and we talked about it being a completely dissociated state. Yes, because the people who accompanied us were crying, the interpreter was crying, and talking about how good the psychologist training is, that gives you something […] and at home, emotionally, what was said started to show. And somehow it started to sink in more, what it really meant. (core team member)

…it was like a delirium… (core team member)

The students also experienced the emotional difficulties of the refugees’ status:

… how insecure they feel, and how they don’t know what tomorrow will bring […] it was very difficult to react well to that and to keep that feeling, that feeling that yes, this is reality. (student)

The language barrier caused particular stress among both core team members and students:

…it was a very big contrast when I went to Kazy (a university program organized in Kazinczy Street) one time, where there were all Ukrainian-speaking families. And it was a much more difficult situation there, and it was a big contrast to the fact that I could get along with 90% of the people I met in the shelter outside the city, and that I suddenly got along with almost nobody there, so it was a completely different situation. (student)

Personal experiences in difficult situations generated different ways of coping. The time spent traveling from the shelter to Budapest and the shared meetings, for example, provided a breathing space and opportunities for unburdening. In particular, the drive from the shelter outside Budapest back into the capital was a time to reflect on things that had happened. The ability to reframe situations and be flexible was another coping mechanism. Meeting the refugees was presented as an encounter with a parallel reality, after which “we had to get back to ourselves.”

For me, the ride back to Pest was always a very good breathing space. So, for me, these drives were very, very important. (student)

And then it usually helped, I think, to tell each other afterwards, obviously anonymously. And then, in […] hindsight, looking back on the day, to have somebody say wow, that’s a really difficult situation. (student)

The helpers had to come to the emotional realization that they could not offer complete and perfect help; they could only be “good enough” helpers and find satisfaction in what they were able to offer at that particular moment:

…because there are people in such a very, very vulnerable situation that you do whatever is possible, and then you say that this is good for now. (core team member)

### 4.4. The professional level

Both the core team members and the students were involved as professionals, and the situation contributed to their professional development. There was much to learn from the facilitation process:

I didn’t know if it was going to work, because maybe they won’t play with us and they’ll surprise us. Then everybody gets to the house […] and the boys and the girls and everyone, and I learn to be very open to see what is going to happen, because sometimes this is activity work. […] It’s flexibility… (core team member)

The experience of working with refugees had an impact on the helpers professionally:

Otherwise, I feel that this more flexible attitude has been extended to the rest of my work. So that it is reflected here in my therapeutic work. And I feel it is a good development. It has been good for me. (core team member)

The students reported good learning experiences and the strengthening of their professional identity:

…from a professional point of view, that any kind of field experience is hugely important I think, and whatever it is, I get that, so it’s not just that we’re learning in the classroom but that we can use that knowledge in some way. (student)

Core team members experienced uncertainties that affected the way they operated professionally:

We actually went into this as lay people, we are not social workers, that’s for sure. […]. We were not primarily refugee specialist providers. We started doing research because that was our field. (core team member)

There was definitely avoidance of traumatization in the professional approach, which was accompanied by constant professional self-reflection:

It makes you think a lot about whether we did it right, whether our colleagues did it right, what happened, so these are anxiety-provoking experiences. (core team member)

The strengthening of professional consciousness was apparent among the psychology students. In the new situation, the familiar differences in status (i.e., the student–teacher relationship) disappeared and a sense of equality was reinforced:

I really learnt a lot about what it is like to have a professional collaboration where everyone’s opinions and ideas matter, and it was very inspiring. (student)

The situation also presented a professional model in a shared field, which was likewise a learning opportunity:

It was possible to work well with professionals, to brainstorm together, and to create such a good professional environment. That was a very important part for me, to see a good model for that. (student)

One aspect of the core team’s professional approach was their sense of responsibility, which they experience in their work in general and which likewise played an important role when working with the refugees:

I think that I try to take responsibility in my work, and I have a sense of responsibility for the children, but it’s just that there are different levels of responsibility, for example, I think about things like the organization of social care, or why they are in that shelter, or how they go to kindergarten or school. (core team member)

### 4.5. Between the personal and professional

There was constant uncertainty at both personal and professional level due to the complexity of the situation, with a marked oscillation between the personal and professional spheres in several areas. The “in-between” category represents the transition between the personal and the professional, which is what made the facilitation process particularly stressful, together with the approach that was adopted as a result.

Identifying personal and professional boundaries emerged as one of the most pronounced strains in this situation. It was difficult for the helpers to balance the time they devoted to the refugees with their family and work commitments. Pushing their professional boundaries also meant having to reinvent themselves in unfamiliar environments. They found themselves performing several roles simultaneously, with an unprecedented patient–therapist relationship in which everyone was more emotionally vulnerable, making it particularly hard to establish emotional boundaries:

I have to set boundaries, how much of this I allow into my life…family trauma…everyone is watching this, and no one is doing anything… I work with different boundaries…the little girl is cuddling…difficult wobble, I set boundaries…how long do my feelings last and where do theirs start… (core team member)

Alongside the roles of psychologist, doctor, and compassionate individual, there was also the role of social worker, and it became difficult to draw the line between these roles when helping the refugees. The students were also conscious of this difficulty:

we got in the car, the boundaries back and forth, it was clear we were buying them clothes. (core team member)

…we also learnt about boundaries, how we should have boundaries, but I feel that in this helping role it was different having boundaries than if I was meeting someone say once a week, and the boundaries were not so clear. (student)

I always find it difficult to work out or draw my boundaries between what I can do now and what I can’t do anymore. (student)

Boundaries were also apparent in terms of putting aside the caution that had become the norm during the Covid period and allowing the needs of the refugees to come first:

I was told that someone had a fever, one of the young children. Remember how we looked at each other? And seriously. And I think there would have been a turning point like that at another time, but… “Okay. So, he’s got a fever. Let’s go in.” (core team member)

There was also uncertainty surrounding the helpers’ professional status. The situation called for roles that had not previously been part of their everyday lives:

…we actually went into this as lay people, we are not social workers, that’s for sure. And it’s very much part of the work […]. We were not primarily refugee specialist care providers. (core team member)

…it makes you think a lot about whether we’re doing it right, whether we’re doing it right, whether we’re doing it right, what’s happened, so it’s a distressing experience. (core team member)

The situation itself often concealed a great deal of uncertainty, which was in itself stressful:

And when we were preparing for chaos, say after the last time, everything went smoothly. So it was total uncertainty. (core team member)

I didn’t know if it was going to work, because maybe they won’t play with us, and they’ll surprise us, then everybody gets to the house and they become the main thing, what they want to do with the dogs, and the boys and the girls and everyone, and I learn to be very open, to see what is going to happen, because sometimes this is activity work. (core team member)

As a way of coping with the uncertainties, both the core team members and the students were able to defend themselves in different ways. Their professional knowledge emerged as a resource on which they were able to draw:

…working within that, we did everything we could to make it good, and what a shame that it’s not a fit that’s good for me, but I could reach back there to therapies, say a therapeutic process, where, in a supportive presence, that’s how it is… (core team member)

…so, it’s totally different. I don’t know, learning it from a textbook, or the development of a disadvantaged child and the bonding that happens, and it’s completely different to experience it in person, and that was such a very important moment, for example, when a little girl came to me after two minutes of knowing me, and she hugged me very, very much and made me love her very much, and then I didn’t even reflect on it for a moment, and then afterwards, I don’t know, half an hour later it hit me that, oops, we learned about that, why this can be or why this happens. (student)

Self-reflection and an awareness of “wins” at personal and group level likewise helped them to cope with the uncertainties:

Because for me, for example, from this question of “Am I good?” or “Am I helping enough?” that I experienced, a big lesson was that I’m not good because […] you give it your all and everything… because I’m giving it all just to burn out all these reserves, but from knowing that I can literally […] think with the others and that will not only be good for me anyway, but it will also make the goal easier to achieve, it will give these families security, and I’ve certainly learned that in this semester, by working together. (student)

I was also trying to remind myself that I was not doing this out of goodness because I was taking a lot from it… (core team member)

But I think we learn about each other’s character and we are not alone. That is for me a fantastic feeling, that whenever… everybody’s very sensitive and whenever a new topic arises then we react on it, and that’s so good. (core team member)

Group members reflected about each other and helped one another to be together in their different roles, complementing each other, marking boundaries, and dividing their efforts:

…I may have been the “mother,” but XY was a hard-headed “father.” (core team member)

…when you and the team just said go away for a weekend and having four days when you have nothing to do, and she took my duties and for me it’s very important that I feel accepted in this way …Because… I feel accepted. (core team member)

## 5. Discussion

To the best of our knowledge, the present study is the first to reflect on the possibility of a research group becoming an aid group in the wake of the outbreak of the war in Ukraine.

Voluntary work began in early March, immediately after the start of the war in Ukraine, and volunteer assistance in one of the projects is ongoing, maintained by core team members. The core team and students participated in two separate focus groups at the end of October, where participants recalled their experiences of volunteering in a semi-structured interview. The volunteers were all women. The content of the focus groups was analyzed using consensual content analysis. Since core team members were involved in the organization of the study and in the content analysis, we employed a postpositivist/constructivist epistemological inductive methodological approach. The non–core team members involved in the process were skilled in the use of qualitative methodology, being psychology graduates with a wealth of experience in intercultural counselling.

Based on the consensual content analysis, five main categories of dynamically interconnected and interdependent themes emerged. The evolution of the helping process shared common features with the four phases of evolution described at the beginning of the present paper (26), although in our case different aspects were emphasized. In the context of our involvement, the helping process evolved as follows: (1) the emergence of emotional internal pressure with several background factors, including personal fears and anxieties, fear for own family members, and emotional identification with the refugees; (2) the incorporation of the help recipients’ point of view, with the emergence of a sense of responsibility, the expression of loyalty, and the prioritization of the recipients’ perspective; and (3) professional and personal adaptation to the situation. In phase 3, the stresses and strains that this adaptation entailed became apparent, along with its powerful impact on professional identity.

The five categories (context; the help recipients’ perspectives—sensitive responsiveness; the personal level; the professional level; and the level between the personal and professional) were the same for the core team and the students, although with differences in content, mainly in the *professional level* and *between the personal and professional* categories. The students relied on and learned from the core team members and participated in the assistance for a limited period, which was recognized as one of their university placements. The students were presumably more emotionally protected due to the experience of the core team and the limited timeframe. The students’ involvement in the assistance contributed to the development of their professional identity, which can be counted as a professional gain. Similarly, the core team members’ existing professional experience was enhanced and their professional identity was likewise developed through volunteering. These experiences are in line with research indicating professional development through volunteering (42–44).

The complex helping processes of the research group and student volunteers evolved through the five categories from *context* to *the level between the professional and personal*. The context emerged from the experience of growing internal tension. The research group’s earlier research themes and engagement with mental health were quickly integrated into the members’ work as helpers. Their attitudes as researchers took a back seat as they deliberately avoided burdening the refugees. In the case of the research group members, previous experience of fieldwork facilitated a rapid transition to the role of helpers. Their practical experience contributed to the present research, and vice versa. Being both practitioners and researchers not only allows for such rapid shifts but also provides an opportunity for serendipitous encounters in which practical work helps to bridge the gaps left by theory (45). This combination may be most relevant in mental health studies, where the attitudes associated with relationships are a very important aspect that affects both the helpers and the helped (45).

The helpers’ identification with the predominantly female and child refugees contributed to the inclusion of the help recipients’ perspective, with sensitive responsiveness playing an important role in creating a safe atmosphere for the refugees. The inclusion of the recipients’ perspective and the attitude of sensitive responsiveness can be paralleled with the patient-centered approach, which is considered effective in the context of patient care (46) and which is closely related to empathy (47). We would also like to draw a parallel with the concept of sensitive responsiveness in the sense used in attachment theories. Sensitive responsiveness describes the ability of parents to accurately identify and respond to a child’s emotional states in a timely and appropriate manner (48). Responsive attitudes send the message that an individual cares about another’s needs, is keen to be more sensitive to them, and wants to contribute to the development of trust and thus a safe atmosphere (49, 50). Patient-centered care involves a humanistic attitude and empathy in Rogersian terms and implies that the helper takes on the perspective of the helped (51, 52). However, this attitude is challenging and demands a great deal of effort on the part of the helper (53). Putting the hope of recovery and well-being at the center of the helper’s perspective contributes to improving the quality of mental health (54). The core team and the students intuitively assumed this to be represented by a sensitive, responsive attitude and empathy.

At a personal and professional level, stressful and demanding situations arose for both the core team and the students. In line with the literature, stresses emerged of a kind that can be considered as psychological traumatization (or vicarious traumatization), such as emotional identification, helplessness, the presence of the traumatized refugees, and constant uncertainty due to the situation (31, 55, 56). Vulnerability to vicarious traumatization is increased by various factors, including female gender and employment in the mental health sector, both of which were relevant in the present case (57). The ongoing search to identify personal and professional boundaries that emerged in the *between the personal and professional* category reflects the specificity of working with refugees, the main areas of strain, and the preventive possibilities. As shown by studies involving counsellors working with refugees, such work requires approaches that go beyond the usual boundaries of providing help: It involves many uncertainties and a blurring of professional and personal boundaries (58, 59). Ongoing personal and group-level self-reflection and the perceived satisfaction obtained from volunteering were also apparent in the “in-between” category as protective factors that contributed to emotional coping. This is likewise in line with the international literature, where ongoing supervision and peer support within the group has been shown to contribute to the helpers’ resilience to psychological trauma (60–63). As mentioned in the introduction to the present paper, professional development, in the form of the gains and satisfaction obtained from volunteering, can be included here as a protective factor (44). Feelings of guilt are often present but vary considerably from individual to individual in a difficult situation of this kind, since adaptive and early maladaptive schemas and coping strategies operate differently among the participants (64–66). Mirović et al. (67) described how early maladaptive schemas, such as self-sacrifice, devaluation, and ruthlessness, which lead to exhaustion in crisis situations, are often to be found in mental health professionals. It is important that helping professionals address this fact in crisis situations such as the war in Ukraine, as they often put their own mental health at the forefront and even feel guilty if they do not (67).

As the facilitators in our study were all women and were providing voluntary assistance mainly to women and children fleeing from Ukraine, it is important to highlight that female attitudes and identification with women refugees may have been a key factor in the process. Several studies have highlighted differences in attitudes among male and female helpers and in the difficulties that arise when working with traumatized people (68–70). The shift from research to the provision of help was presumably also facilitated by the ongoing fulfilment of multiple roles by the women (in relation to family, childcare, professional development, learning, and work). Several members of the core team are also mothers, and all of them have been working with children and families for many years. The students are all enrolled at the Department of Developmental and Clinical Child Psychology and most are training to work as psychologists with children and families. Since the refugees from Ukraine are mostly women and children, a potential area of research in the future would be to investigate the motivations and difficulties experienced by male helpers, which are not represented in our work.

Our conclusions must be interpreted in the light of the limitations of the present study, which correspond to the general limitations of qualitative research and include the small number of subjects and the lack of statistical calculations and approaches. A further limitation of our study is that it did not use mixed methods as a quantitative approach or add additional qualitative methods that would have provided further analytical possibilities. In the present case, the qualitative research reflects the situation of women researchers in a specific situation in the new and as yet under-researched field of voluntary work with refugees from the war in Ukraine.

In summary, we highlight the following findings: (1) The five categories that emerged from the consensual content analysis illustrated the development of the helping process in the present case, as well as revealing what made the provision of voluntary help burdensome. (2) The helping process described here demonstrates the way in which female helpers operate, since the helpers were all women and were helping mostly women and children. (3) In addition to experiencing psychological stress, the volunteers benefited from their participation in the process in terms of their professional development. (4) Thanks to group members’ experience of practical work, the research group was quickly able to transform itself into a support group.

Learning from our experience, we could suggest that when a research team is transformed into a helping team, it is very important to take into account the workload of the team members. These should be discussed openly and transparently. It is important to prepare for emotional stress by making group members aware of the difficulties they may encounter. It is important to maintain team meetings where, in addition to practical tasks, there is an opportunity to express emotional difficulties. The use of professional supervision can also be very useful. It is also recommended that during the support process, participants keep a memo of what happened and reflect on their own feelings. This process can be useful from many points of view. On the one hand, it can serve to maintain mental health by helping to process the events, and on the other hand it can be useful material for formulating further research questions in case the research team would like to link to research aspects of the topic in the future.

## Data availability statement

The raw data supporting the conclusions of this article will be made available by the authors, without undue reservation.

## Ethics statement

The studies involving human participants were reviewed and approved by Research Ethics Committee (REC) of the Faculty of Education and Psychology of ELTE. The patients/participants provided their written informed consent to participate in this study.

## Author contributions

XR-V, JB, and LANL were responsible for the design of the study and the preparation of the manuscript. XR-V and ZK were responsible for the design of the study method and the qualitative data collection. XR-V, EF-B, LANL, BFF, and ZK were actively involved in the analysis of the interviews. JB, EF-B, BFF, XR-V, NK, LOH, and AB were responsible for the professional planning of the volunteer work. JB, AB, and NK were responsible for continuously monitoring it. JB, EF-B, BFF, XR-V, NK, LOH, and AB actively participated in the volunteer work. XR-V drafted the manuscript. EF-B, JB, and LANL language proofread it. All authors contributed to the manuscript revision, and read and approved the submitted version.

## Conflict of interest

The authors declare that the research was conducted in the absence of any commercial or financial relationships that could be construed as a potential conflict of interest.

## Publisher’s note

All claims expressed in this article are solely those of the authors and do not necessarily represent those of their affiliated organizations, or those of the publisher, the editors and the reviewers. Any product that may be evaluated in this article, or claim that may be made by its manufacturer, is not guaranteed or endorsed by the publisher.
